# Correction: Fruity, sticky, stinky, spicy, bitter, addictive, and deadly: evolutionary signatures of metabolic complexity in the Solanaceae

**DOI:** 10.1039/d2np90030k

**Published:** 2022-09-20

**Authors:** Paul D. Fiesel, Hannah M. Parks, Robert L. Last, Cornelius S. Barry

**Affiliations:** Department of Biochemistry & Molecular Biology, Michigan State University East Lansing MI 48824 USA; Department of Plant Biology, Michigan State University East Lansing MI 48824 USA; Department of Horticulture, Michigan State University East Lansing MI 48824 USA barrycs@msu.edu

## Abstract

Correction for ‘Fruity, sticky, stinky, spicy, bitter, addictive, and deadly: evolutionary signatures of metabolic complexity in the Solanaceae’ by Paul D. Fiesel *et al.*, *Nat. Prod. Rep.*, 2022, **39**, 1438–1464, https://doi.org/10.1039/D2NP00003B.

The authors regret that the acylation positions on the sugar backbone of the acylsugars depicted in Fig. 4 were drawn incorrectly in the article and that the reaction of SpASAT2 was omitted. A corrected version of Fig. 4 is shown below. Spelling of the Latin name of the wild tomato was also corrected.

**Fig. 1:**
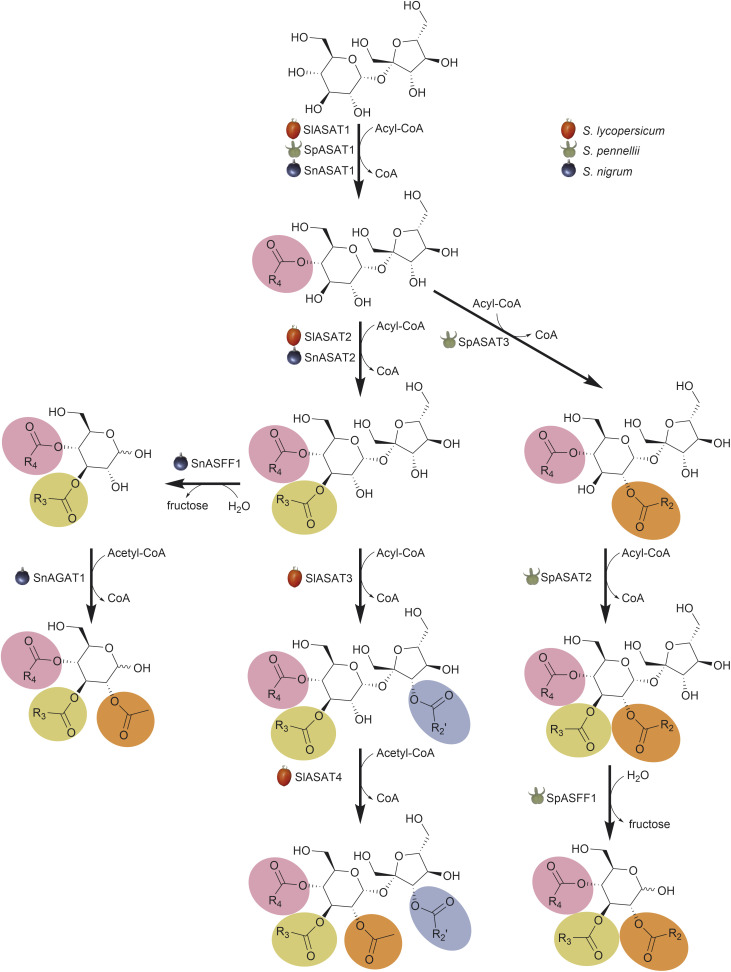
Acylsucrose and acylglucose pathway diversity in *Solanum* species. The acylsucrose and acylglucose biosynthesis pathways for *S. nigrum*, *S. lycopersicum* and *S. pennellii*. All three biosynthetic pathways begin by acylating sucrose.^24,63,64,68,72^ Sequential acylations produce tetraacylsucroses, triacylsucroses, and diacylsucroses for *S. lycopersicum*, *S. pennellii*, and *S. nigrum*, respectively. *S. pennellii* triacylsucroses and *S. nigrum* diacylsucroses are cleaved by ASFF enzymes to form triacylglucoses and diacylglucoses, respectively.^68,72^*S. nigrum* diacylglucose is acetylated by SnAGAT1 to form a triacylglucose.^72^ ASAT, acylsucrose acyltransferase; AGAT, acylglucose acyltransferase; ASFF, acylsugar fructofuranosidase; CoA, CoenzymeA.

The Royal Society of Chemistry apologises for these errors and any consequent inconvenience to authors and readers.

## Supplementary Material

